# Anticancer Activities and Mechanism of Action of Nagilactones, a Group of Terpenoid Lactones Isolated from *Podocarpus* Species

**DOI:** 10.1007/s13659-020-00268-8

**Published:** 2020-10-09

**Authors:** Christian Bailly

**Affiliations:** OncoWitan, 59290 Lille (Wasquehal), France

**Keywords:** Cancer, Natural products, Mechanism of action, Molecular target, Terpenoids

## Abstract

Nagilactones are tetracyclic natural products isolated from various *Podocarpus* species. These lactone-based compounds display a range of pharmacological effects, including antifungal, anti-atherosclerosis, anti-inflammatory and anticancer activities reviewed here. The most active derivatives, such as nagilactones C, E and F, exhibit potent anticancer activities against different cancer cell lines and tumor models. A comprehensive analysis of their mechanism of action indicates that their anticancer activity mainly derives from three complementary action: (i) a drug-induced inhibition of cell proliferation coupled with a cell cycle perturbation and induction of apoptosis, (ii) a blockade of the epithelial to mesenchymal cell transition contributing to an inhibition of cancer cell migration and invasion and (iii) a capacity to modulate the PD-L1 immune checkpoint. Different molecular effectors have been implicated in the antitumor activity, chiefly the AP-1 pathway blocked upon activation of the JNK/c-Jun axis. Nag-C is a potent inhibitor of protein synthesis binding to eukaryotic ribosomes and inhibition of different protein kinases, such as RIOK2 and JAK2, has been postulated with Nag-E. The literature survey on nagilactones highlights the therapeutic potential of these little-known terpenoids. The mechanistic analysis also provides useful information for structurally related compounds (podolactones, oidiolactones, inumakilactones) isolated from *Podocarpus* plants.

## Introduction

**Fig. 1:**
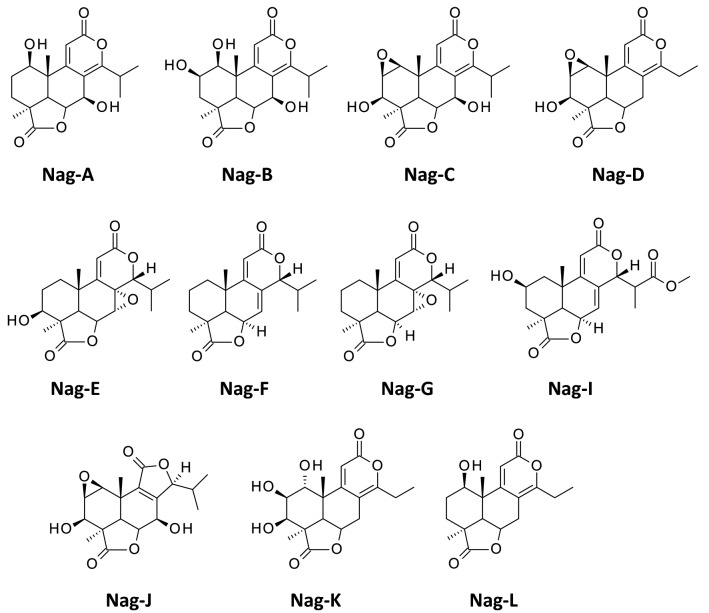
Chemical structure of the nagilactones. The structure of Nag-H could not be identified

The nagilactones refer to a group of bioactive terpenoids initially isolated from the plant *Podocarpus nagi* (Thunb.) Zoll. et Moritz. in Japan in the late 1960s [1, 2]. *P. nagi* belongs to the Podocarpaceae, the second largest family of conifers [3]. Over the past 60 years, a dozen of nagilactones, named from A to L (Fig. 1), have been isolated as well as a variety of structural analogues, from various *Podocarpus* species, such as *P. nivalis*, *P. gracilior*, *P. elongatus*, *P. hallii*, *P. neriifolius*, *P. fasciculus*, *P. nakaii* and *P. falcatus*, using either the seeds, roots, twigs, barks or leaves of the plants [4–10]. Novel *Podocarpus* terpenoids are regularly reported and studies with *P. nagi* itself continue to reveal the presence of different diterpenoids, like nagiol A and dihydroxylambertic acid, identified in recent years [11, 12]. Various epoxy-nagilactones and mono- and di-glycoside derivatives have also been found [13–15].

**Fig. 2:**
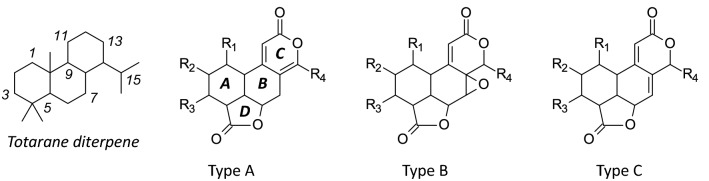
The three chemical types of tetracyclic nagilactones. They differ by the organization of the conjugated lactone system in the *B*–*C* ring moiety. The numbering of the totarane diterpene nucleus is indicated

Nagilactones (Nag) and related tetracyclic lactone compounds are usually classified into three structural types depending on the nature of the conjugated lactone system in the B–C ring moiety. Type A compounds present an a-pyrone [8(14),9(11)-dienolide] unit. Type B compounds bear a 7a,8a-epoxy-9(11)-enolide structure. Type C compounds correspond to a 7(8),9(11)-dienolide structure (Fig. 2). The classification, first proposed by Hayashi and Matsumoto [16], remains used to classify these lactone products, nagilactones but also podolactones [17]. Bioactive compounds can be found in each group, such as Nag-C (type A), Nag-E (type B) and Nag-F (type C) (Table 1). A noticeable exception to the rule is Nag-J (Fig. 1) which has a unique bisnorditerpene dilactone and is the only nagilactone derivative whose C-ring is a 5-membered ring lactone [18].

**Table 1:** Nagilactones (Nag)

Compounds	CID number^a^	Formula and molecular weight (g/mol)	Type^b^ (A–B–C)	References
Nag-A	3084328	C_19_H_24_O_6_ 348.4	A	[1]
Nag-B	3084329	C_19_H_24_O_7_ 364.4	A	[1, 68]
Nag-C	319648, 72505, 319648, 442063, 138113851, 5458434,	C_19_H_22_O_7_ 362.4	A	[1, 4, 68]
Nag-D	3084330	C_18_H_20_O_6_ 332.3	A	[1, 68]
Nag-E	72504, 457159, 54607199	C_19_H_24_O_6_ 348.4	B	[2, 68]
Nag-F	181498, 100002	C_19_H_24_O_4_ 316.4	C	[62, 63, 68, 69]
Nag-G	71300384	C_19_H_24_O_5_ 332.4	B	[32, 70]
Nag-I		C_18_H_22_O_7_ 350.4	C	[10]
Nag-J		C_18_H_21_O_7_ 349.3	–	[18]
Nag-K		C_18_H_21_O_7_ 349.2	A	[28]
Nag-L		C_18_H_23_O_5_ 319.2	A	[28]

^a^Compound Identity number (PubChem CID). Nag-I/J/K/L are not listed in PubChem

^b^The structural type refers to Fig. 2. Compound Nag-J corresponds to a different structure, with a five-membered lactone C-ring, as shown in Fig. 1. The structure of Nag-H could not be identified. Nagilactones I–L are not inventoried in PubChem

**Fig. 3:**
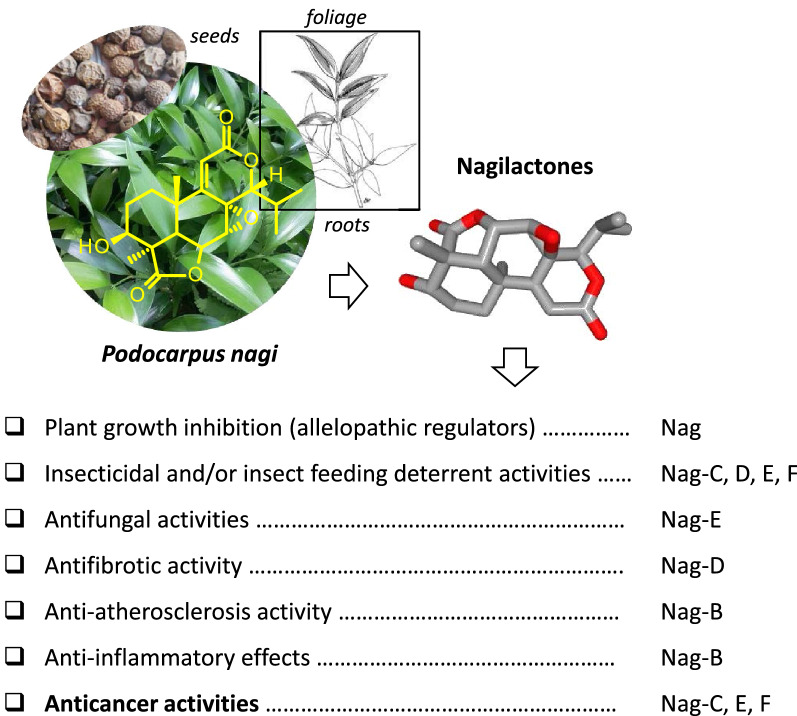
The various biological properties of nagilactones, principally isolated from the seeds, roots, foliage of *Podocarpus nagi* [67] and other *Podocarpus* species

Under physiological conditions, nagilactones serve as plant growth inhibitors (allelopathic regulators) or anti-herbivory substances [19]. In addition, diverse pharmacological properties have been described, as illustrated in Fig. 3. Nagilactones C, D, and F cause insect feeding deterrent activity ultimately coupled to an insecticidal activity [8, 20]. Nag-C has shown a high insecticidal activity against second-instar nymphs of the predatory stink bug *Eocanthecona furcellata* [21]. The drug is toxic also to housefly larvae (*Musca domestica* L.), inhibiting larval growth, maturation to pupae, and emergence of adults [4]. Similarly, Nag-D was found active against different insects, such as the apple moth *Epiphyas postvittana* and the codling moth *Laspeyresia pomonella* [22, 23].

Modest antifungal activities have also been found [24]. But the interest for nagilactones essentially comes from their cytotoxic and anticancer potential, which was recognized very early on, with the characterization of the antitumor potential of nagilactones B–E as early as 1975 [25]. But it is only recently that the mechanism of action of anticancer nagilactones has been elucidated, at least partially. Recent studies have brought essential information to apprehend the mode of action of the most potent compounds in the series, Nag-C and Nag-E. A review of the pharmacological properties of nagilactones is offered here, with a focus on the anticancer activities and the underlying mechanisms, target and signaling pathways implicated.

## Anticancer Activities of Nagilactones

Several studies have reported anticancer activities with nagilactones. Nag-C has revealed marked antiproliferative effects against two cancer cell lines in vitro: human HT-1080 fibrosarcoma and murine colon 26-L5 carcinoma, with ED_50_/IC_50_ in the 3–6 mM range [6]. A roughly equivalent potency was reported with other cancer cell lines, such as the breast cancer cell line MDA-MB-231, the gastric cancer cell line AGS and the cervical cell line HeLa, with IC_50_ of 3–5 mM [26]. In the same study, Nag-F and Nag-G were found to be more cytotoxic than Nag-C, with IC_50_ around or below 1 mM against the same human cancer cell lines [26]. The structurally related compounds inumakilactone B and podolactone E were equally potent at inhibiting the growth of different cancer cell lines in vitro, with IC_50_ in the 1–5 mM range also [27]. Not all nagilactones are cytotoxics. For example, nagilactone K was found to be inactive against the ovarian cancer cell lines A2780 and HEY, with IC_50_ > 10 mM, whereas nagilactones A, B, C, D and G were active, with IC_50_ in the range 2–10 mM. In this in vitro study the most potent compound was Nag-D and the derivative 2β-hydroxynagilactone L, which was characterized as a potent inducer of autophagic cancer cell death [28].

**Fig. 4:**
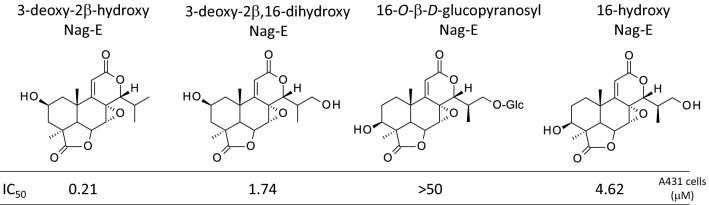
Structure–anticancer activity relationships in the Nag-E series. The indicated IC_50_ values (mM) correspond to the compound concentrations required to reduce proliferation of human epithelial carcinoma A431 cells by 50%, as described by Zheng et al. [15]

A cytotoxic evaluation has also been performed in the frame of a study of diverse natural products isolated from the plant *P. fasciculus*. In this work, Nag-C was found to be three times more active against the colon tumor cell line DLD compared to the human oral epithelium carcinoma KB cell line. Nag-C was more potent than the diverse flavonoids isolated from the plant [7]. Another study reported the cytotoxic activities of makilactones and a few nagilactones isolated from *Podocarpus macrophyllus* against P-388 leukemia cells in vitro. In this case, Nag-G was found to be remarkably active (significantly more potent than all makilactones tested), with an IC_50_ of ~ 0.25 mM (0.08 mg/ml) comparable to that measured with the reference anticancer alkaloid camptothecin. Nag-E was also active (IC_50_ = 0.25 mg/ml), but three times less potent than Nag-G [29]. This is consistent with a recent study indicating that Nag-E dose-dependently reduces the growth of human non-small cell lung cancer cells A549 and NCI-H1975, with IC_50_ of 5.2 and 3.6 μM, respectively [30]. The derivative 3-deoxy-2β-hydroxy-nagilactone E (Fig. 4) is also a potent inhibitor of cancer cell growth, 3-to-8-fold more active than its derivative 3-deoxy-2β,16-dihydroxy-nagilactone E (designated B6, [31]) against different cancer cell lines, and much more potent than its glycoside derivative 16-O-β-d-glucopyranosyl-nagilactone E which is totally inactive in vitro [15]. Preliminary structure–activity relationships can be defined in the Nag-E series. Nag-E derivatives with a 2-OH or 3-OH provide cytotoxic compounds but the glycosylation at the 16-OH position leads to a complete loss of activity (Fig. 4). This compound 3-deoxy-2β-hydroxy-nagilactone E, isolated from *P. neriifolius*, is interesting because recently it was found to be active in vitro using ovarian (OVCAR3), melanoma (MDA-MB-435) and colon (HT-29) cancer cell types. The related compound inumakilactone A, which is less active than deoxy-2β-hydroxy-nagilactone E in vitro, was found to be inactive in an in vivo hollow fiber assay using the same cell lines. Unfortunately, the available quantity of 3-deoxy-2β-hydroxy-nagilactone E was not sufficient for in vivo testing [32].

Proofs of anticancer activities of nagilactones in vivo are limited. The initial report of 1975 showed a dose-dependent activity of Nag-C against the P-388 leukemia in mice, with the drug injected daily at 10–20–40 mg/kg intraperitoneally. Nag-E also showed an in vivo activity, but it was more toxic at the highest dose of 40 mg/kg [25]. The best evidence of in vivo activity comes from a recent study with Nag-E in an A549 cell lung cancer xenograft mouse model. The drug, injected i.p. at 10 mg/kg, reduced tumor growth in vivo by 62% and reversed tumor metastasis, without any apparent toxic effect [33].

## Other Pharmacological Activities of Nagilactones

Episodically, different types of pharmacological activities have been reported with nagilactones. Insecticidal activities were initially described, as mentioned above, in addition to antifungal effects. A recent work analyzed the mechanism of Nag-E using the non-pathogenic fungus *Saccharomyces cerevisiae* and showed that the drug induced morphological cell changes (inhomogeneous thickness of the glucan layer and leakage of cytoplasm) and a dose-dependent decrease of de novo synthesis of 1,3-β-glucans. Comparable cell changes were observed using the human pathogenic fungus *Aspergillus fumigatus* [34]. The antifungal activity of Nag-E has also been evidenced using two other fungi: *Candida albicans* and *Pityrosporum ovale* [24, 35, 36].

An anti-inflammatory component has been discussed. The derivative 1-*O*-β-d-glucoside-nagilactone B, isolated from seeds of *P. nagi*, significantly inhibited nitric oxide (NO) production on LPS-stimulated RAW264.7 macrophages, with IC_50_ values of 0.18 mM. The drug inhibited the activity of the transcription factor NFkB, thereby suppressing the expression of inducible NO synthase (iNOS), which is a classical pro-inflammatory mediator [37]. This nagilactone derivative could inhibit the nuclear translocation of NFkB, as observed with sciadonic acid which is another natural product isolated from seeds of *P. nagi* [38].

Atypically, Nag-B was found to present an anti-atherosclerosis activity in a specific mouse model. The drug could ameliorate cholesterol accumulation in macrophages via improving apolipoprotein A-I and HDL-mediated cholesterol efflux [39]. The related compound Nag-D has been shown to exert marked anti-fibrotic activity in a mouse model of bleomycin-induced pulmonary fibrosis. A detailed analysis of the mechanism indicated that Nag-D suppressed the expression of the transforming growth factor-β1 (TGF-β1) and the phosphorylation, nuclear translocation, and transcriptional activation of the protein Smad2 [40]. The drug impact on the TGF-β1/Smad signaling pathway is important, not only because it can explain at least partially the anti-fibrotic activity of Nag-D but it can contribute also to the anticancer effects. For example, inhibition of the TGF-β1/Smad pathway has been invoked to explain the anticancer effects of different natural products such as paenol and oridonin [41, 42]. Therefore, the recently discovered capacity of Nag-D to regulate the fibroblast-to-myofibroblast transition in atherosclerotic mice deserves further attention because the underlying mechanism is similar to that implicated in the epithelial–mesenchymal transition (EMT) which is known to play a major role to increase motility and invasiveness of cancer cells, and thus to promote cancer metastasis. In fact, this mechanism has been proposed recently to explain the anticancer activity of Nag-E in a model of lung cancer. As discussed below, Nag-E reduces the mRNA and protein expression of the receptor TβRI, induced by the ligand TGF-β1 [43].

## Molecular Targets and Mechanisms of Action of Nagilactones

The mode of action of nagilactones is multifactorial, as it is frequently the case with natural products. It is not entirely elucidated, but several key pathways have been implicated in the antitumor activity of the most active compounds, essentially Nag-C, Nag-E and Nag-F. About ten years ago, it was shown that Nag-F was able to inhibit activation of the dimeric transcription factor AP-1 (activator protein 1) induced by the phorbol ester TPA (12-*O*-tetradecanoyl probol-13-ester), with a micromolar efficacy [44]. AP-1, which is in fact a group of transcription factors consisted of four sub-families (Jun, Fos, Maf, and the ATF-activating transcription factors), represents an important modulator in several immune disorders and carcinomas. It is frequently overexpressed in cancers [45] and it plays key roles in the regulation of anti-tumor immune responses [46]. By blocking the AP-1 oncogenic pathway, Nag-F can exert both a direct action on the growth of AP-1-dependent tumors and an indirect immunogenic action via the regulation of immune checkpoints, such as the major PD-1/PD-L1 checkpoint. A seminal role of AP-1 in regulating PD-L1 expression has been evidenced [46]. Interestingly, a recent study has demonstrated that the antitumor compound Nag-E increases PD-L1 expression in lung cancer cells through activation of c-Jun, which is a component of the AP-1 pathway [47]. Nag-E was found to promote PD-L1 expression and exposure on plasma membrane of cancer cells. This phenomenon could be significantly reduced by the knockdown of c-Jun or pharmacological inhibition of c-Jun N-terminal kinases (JNKs), thus placing the JNK/c-Jun axis as an upstream regulator that mediates the action of Nag-E. In fact, Nag-E is known to function as a protein synthesis inhibitor [43], and the ensuing drug-induced ribotoxic stress response can be responsible for the activation of JNK. This has been reported with other natural products inhibitors of protein synthesis (e.g. anisomycin) [48] and remarkably, another compound in the Nag family, 2β-hydroxy-nagilactone L, has also been found to activate the JNK pathway. The pretreatment with a specific JNK inhibitor markedly reduced the activity of this compound and its capacity to induce autophagy, thus also supporting the view that the JNK axis is key to the mechanism of action of these compounds [28].

**Fig. 5:**
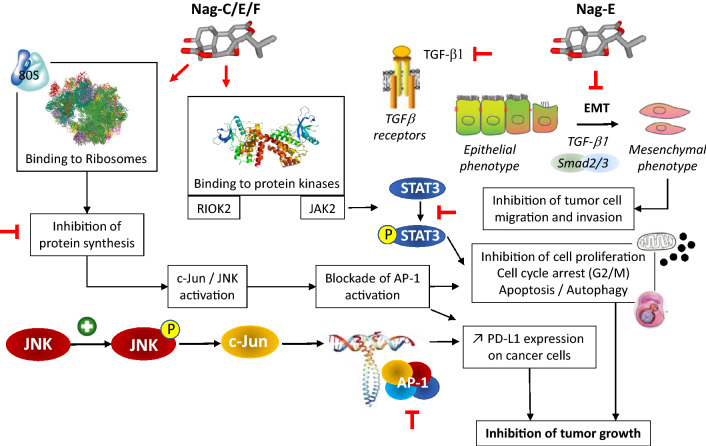
Schematic view of the mechanism of action of antitumor nagilactones (Nag-C–E). Binding of the drug to ribosome leads to protein synthesis inhibition. Binding to the protein kinases RIOK2 and JAK2 has been also reported, leading to inhibition of phospo-STAT3, and then to cancer cell growth inhibition and induction of apoptosis. In addition, Nag-E was found to inhibit epithelial to mesenchymal transition (EMT) of cancer cells, thereby reducing their capacity to migrate and invade tissues (inhibition of metastasis)

**Fig. 6:**
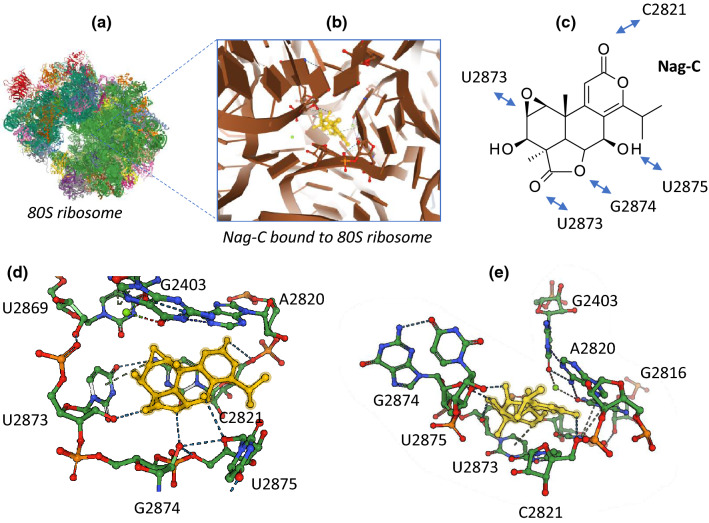
Illustration of Nag-C bound to the 80S ribosome (PDB code U52) [50]. **a** The entire yeast 80S ribosome, **b** a focused view of Nag-C at the heart of the ribosome, blocking the peptidyl transferase center, **c** the molecular contacts (H-bonds and p stacking interactions) between residues of Nag-C and nucleotides of the 25S ribosomal RNA unit, **d** and **e** two views of the drug (in yellow) bound to the ribosomal site, in a cavity formed by the indicated RNA nucleotides

Based on our analysis of the literature on nagilactones, a schematic representation of the mechanism of action of the antitumor nagilactones can be proposed (Fig. 5). The capacity of Nag-E to inhibit protein synthesis in cancer cells has been firmly established, with the efficient dose-dependent inhibition of different short-lived proteins (Nrf2, p21, ATF4) and inhibition of de novo protein synthesis. Similarly, inhibition of protein synthesis has been demonstrated with Nag-C in HeLa cells. In these cervical cancer cells, protein synthesis was decreased by 95% within 10 min of exposure to Nag-C and an active translation was found to be necessary for ribosome dissociation by the drug. Nag-C selectively interferes with translation elongation [49]. This compound was later shown to bind to the 60S tRNA A-site of the eukaryotic ribosome, hindering aminoacyl-tRNA positioning in the peptidyl transferase center (PTC) and thus blocking the peptide bond formation. An illustration of Nag-C bound to the yeast ribosomal PTC is presented in Fig. 6, derived from the crystal structure of the drug bound to yeast 80S ribosome [50]. Nag-C fits well into a narrow cavity at the center of the ribosomal structure, stabilized by several molecular contacts between the lactone units of the drug and the surrounding nucleotides, as shown in Fig. 6. Its mode of action at the ribosome level is comparable to that observed with other naturally occurring inhibitors of protein synthesis like narciclasine, lycorine and homoharringtonine which also present potent anticancer activities [50].

In parallel, a direct binding of Nag-E to an allosteric site of the human RIO kinase 2 (RIOK2) has been proposed based on a molecular docking study. A small hydrophobic shallow cavity in the protein is apparently well adapted to the conformation of Nag-E, thus susceptible to lock the kinase in an inactive conformation [33]. This is at present a hypothesis, that will require an experimental validation, but it is entirely plausible and consistent with the protein inhibition data (Fig. 5). RIOK2 is an atypical serine/threonine protein kinase which regulates ribosomal biogenesis, overexpressed in some cancers, and implicated in the migration/invasion phenomenon through epithelial–mesenchymal transition [51–53]. The potential selective targeting of this kinase by Nag-E is interesting in the field of cancer, and beyond because the kinase is also essential to some parasites [54, 55]. But, at present the targeting of RIOK2 by Nag-E is an in silico proposal, awaiting an experimental validation and this enzyme may not be the only kinase inhibited by Nag-E.

The Nag-E derivative designated B6 (3-deoxy-2β,16-dihydroxy-nagilactone E) has been shown to inhibit the Janus kinase 2 enzyme (JAK2) [31]. It potently inhibits IL-6-induced phosphorylation of JAK2 and thus reduces the phosphorylation of the transcription factor STAT3 which is phosphorylated by JAK2. Interestingly, this compound does not classically bind to the kinase pocket of the enzyme, as it is frequently the case with small molecule inhibitors, but it was found to interact with the noncatalytic FERM-SH2 domain of JAK2. In doing so, B6 preferentially inhibits the growth of cancer cells with overactivated STAT3, like the MDA-MB-231 and MDA-MB-468 breast cancer cell lines, both potently engaged toward apoptosis under treatment with the Nag-E derivative [31].

The treatment of non-small cell lung cancer cells (A549 and NCI-H1975) with Nag-E was found to induce G2/M phase cell cycle arrest through down-regulation of the protein kinase Wee1, the protein Cyclin B1 and reduction of the phosphorylation of cyclin-dependent kinases cdc2 and cdc25C. These effects led to a prominent induction of caspase-dependent cancer cell apoptosis [30]. These effects could well result from the inhibition of the JAK2/STAT3 signaling pathway, as observed with other natural products, notably with the sesquiterpene lactone-based product named dehydrocostus lactone [56] and other plant natural products [57, 58].

Finally, one of the main molecular traits of Nag-E is its capacity to inhibit TGF-b1-induced epithelial-to-mesenchymal transition (EMT), via the suppression of Smad-2/3 activation and nuclear translocation (Fig. 5). In doing so, Nag-E inhibits the migration and invasion of cancer cells [43]. The transforming growth factor (TGF)-β1 is a key driver of EMT, largely implicated in cancer metastasis [59]. EMT inhibition by Nag-E, observed in vitro, likely contributes to the antitumor action in vivo. Altogether, the anticancer action of Nag-E involves three complementary actions: a cytotoxic impact, an anti-metastatic effect, and an immuno-modulatory action (Fig. 5).

## Conclusion

The evergreen tree *P. nagi* (also known as *Nageia nagi*) is widely distributed in Asia, notably in China (竹柏 or Zhubai), Japan (ナギ; nagi or naki) and Vietnam (Kim giao núi đất). It grows also well throughout the southern United States [60]. Various natural products have been isolated from *P. nagi*, notably flavonoids, terpenes, a few steroids and different terpenoid lactones, principally represented by the nagilactones [61]. In fact, nagilactones have been isolated different plants of the genus *Podocarpus* (which contains 94 species) growing all over the world. For example, Nag-I and derivatives were isolated from the specie *P. falcatus*, widely distributed in several African countries [10]. The compounds are usually obtained by extraction from different parts of the plants, but total enantio-selective syntheses have been developed as well, notably for the bioactive compounds such as Nag-F [62–64]. The availability of synthetic products is important to provide an easier access to these complex natural products and the supply of large quantities necessary to permit extended pharmacology and toxicology studies, and later to reach industrial scale supply [65].

Nagilactones are endowed with antifungal, anti-inflammatory, anti-atherosclerosis and anticancer activities, as reviewed here. Recent progresses have been made to better comprehend the mechanism of action of the most potent compounds, in particular Nag-C and Nag-E. The capacity of Nag-E to regulate the AP-1 transcription factor stands as a prime element of the mechanism of action of this compound. The AP-1 pathway is implicated in diverse cellular processes and pathologies. It represents a key target for structurally related compounds such as the inumakilactones [44, 66]. Therefore, understanding the mechanism of action of nagilactones will help to better comprehend the mode of action of structurally related metabolites isolated from *Podocarpus* plants, such as the podolactones A–D, podolide, sellowin A–B, oidiolactones A–D, CJ 14515, hallactone and inumakilactones A–B.

In conclusion, this review provides an insight into the mode of action of nagilactones, with a focus on their antitumor activity. Nag-C, E, and F represent interesting archetypes from which more active derivatives could be designed. They show antitumor activities in vitro and in vivo, resulting from their capacity to inhibit cancer cell growth and proliferation, to restrict cancer cell migration and invasion and to modulate immune checkpoints. The AP-1 pathway is as the heart of their mechanism of action and inhibition of protein synthesis represents an upstream element of their activity. Further studies are required to better define their molecular targets and to better characterize their antitumor activities in animal models. To date, there is practically no available information about the safety, bioavailability and pharmacokinetic parameters of nagilactones. This family of natural products warrant further investigations.

## Abbreviations

Nag: Nagilactone
